# Susceptibility of Clostridium difficile Isolates of Varying Antimicrobial Resistance Phenotypes to SMT19969 and 11 Comparators

**DOI:** 10.1128/AAC.02000-15

**Published:** 2015-12-31

**Authors:** Jane Freeman, Jonathan Vernon, Richard Vickers, Mark H. Wilcox

**Affiliations:** a Microbiology, Leeds Teaching Hospitals Trust, University of Leeds, Leeds, United Kingdom; b Healthcare Associated Infections Research Group, Leeds Institute for Biomedical and Clinical Sciences, University of Leeds, Leeds, United Kingdom; c Summit Therapeutics, Abingdon, Oxfordshire, United Kingdom

## Abstract

We determined the *in vitro* activity of SMT19969 and 11 comparators, including metronidazole, vancomycin, and fidaxomicin, against 107 C. difficile isolates of different antimicrobial resistance phenotypes. Fidaxomicin and SMT19969 were the most active. The fidaxomicin and SMT19969 geometric mean MICs were highest in ribotypes known to show multiple resistance. Coresistance to linezolid and moxifloxacin was evident in ribotypes 001, 017, 027, and 356. The high-level ceftriaxone resistance in ribotypes 356 and 018 was location linked.

## TEXT

*Clostridium difficile* infection (CDI) is a significant cause of nosocomial diarrhea and a continuing burden on health care resources (1). Most antimicrobials have been associated with CDI cases at some point, including the treatment agents vancomycin and metronidazole (Clostridium difficile Ribotyping Network [CDRN] for England and Northern Ireland 2011-2013; https://www.gov.uk/government/publications/clostridium-difficile-ribotyping-network-cdrn-report). CDI recurrence is common following conventional antimicrobial treatment and is associated with reduced gut bacterial diversity (2). Treatment options remain limited, despite the introduction of fidaxomicin for CDI; therefore, development of novel antimicrobial agents, particularly ones with a lower potential for gut microbiota depletion, is needed. SMT19969 is a novel antimicrobial with potent *in vitro* activity against C. difficile (3) but limited activity against gut microflora (4). We investigated the activity of SMT19969 and 11 comparators, including predisposing and treatment agents, against C. difficile isolates of different antimicrobial resistance phenotypes.

A panel of 107 C. difficile isolates was selected from a collection assembled during the *Clos*ER study (5) by permission of Astellas Pharma Europe. Clinical isolates were collected between July 2011 and April 2013 and were selected to maximize the diversity of antimicrobial resistance phenotypes. The susceptibilities of C. difficile isolates to metronidazole, vancomycin, fidaxomicin, rifampin, moxifloxacin, clindamycin, imipenem, chloramphenicol, tigecycline, SMT19969, linezolid, and ceftriaxone were determined using a Wilkins-Chalgren agar incorporation method (5, 6). The MIC was defined as the lowest dilution at which growth was completely inhibited or at which only single colonies remained.

The MIC results for each isolate were designated susceptible (S), intermediately resistant (I), fully resistant (R), or reduced susceptibility (RS) according to the breakpoints defined in Table 1. The breakpoints were established according to the Clinical Laboratory Standards Institute (CLSI), the European Committee on Antimicrobial Susceptibility Testing (EUCAST), or published data. Each result was assigned a score (S = 0, I = 1, and R = 2). A cumulative resistance score (CRS), based on susceptibility to each of the 11 antimicrobials tested, was generated for each isolate. Thus, an isolate that was fully susceptible to 6, intermediately resistant to 2, and resistant to 3 antimicrobials would generate a score of 8 (0 + 0 + 0 + 0 +0 + 0 + 1 + 1 + 2 + 2 + 2).

**TABLE 1:** Susceptibility of 107 *C. difficile* isolates to SMT19969 and 11 comparators

Parameter	Results for drug (breakpoint):											
	SMT19969	Fidaxomicin (S < 1; RS > 1^5^)	Metronidazole (S < 2; I = 4; R > 8^5^)	Vancomycin (S < 2; I = 4; R > 8^5^)	Rifampin (S < 0.002; I = 0.003–16; R > 32^5^)	Moxifloxacin (S < 2; I = 4; R > 8^5^)	Clindamycin (S < 2; I = 4; R > 8^5^)	Imipenem (S < 4; I = 8; R > 16^5^)	Chloramphenicol (S < 8; I = 16; R > 32^5^)	Tigecycline (S < 0.2^5^; RS > 0.25^5^)	Linezolid (S < 4; R > 4^11^)	Cetriaxone (S < 16; I = 32; R > 64^14^)
Geometric mean	0.07	0.04	0.41	0.80	0.03	9.81	19.00	4.00	5.97	0.04	5.19	58.18
MIC_50_	0.03	0.06	0.5	1	0.002	16	16	4	4	0.03	4	64
MIC_90_	0.125	0.125	2	2	32	32	128	8	16	0.06	16	128
Range	0.015–0.5	0.004–0.125	<0.125–2	0.5–8	<0.001−>32	1–128	2–128	1–16	4−>256	0.03–0.25	2–32	16−>256
%S		100.00	100.00	100.00	70.64	31.19	7.34	73.39	84.40	99.07	85.32	6.42
%I		0.00	0.00	0.00	2.75	0.92	6.42	22.94	11.01	0.93	0.92	33.03
%R		0.00	0.00	0.00	26.61	67.89	86.24	3.67	4.59	0.00	13.76	60.55

Fidaxomicin was the most active agent, followed by SMT19969, with similar geometric mean (GM) MICs (0.04 mg/liter versus 0.07 mg/liter, respectively) (Table 1) and with no evidence of resistance to either agent (Table 1). Fidaxomicin (GM MIC of 0.04 mg/liter) was 10- and 20-fold more active than metronidazole (GM MIC of 0.41 mg/liter) and vancomycin (GM MIC of 0.80 mg/liter), while SMT19969 (GM MIC of 0.07 mg/liter) was 6- and 11-fold more active, respectively. The MICs of both fidaxomicin and SMT19969 were comparable to those observed previously (3, 5, 7, 8). Although the fidaxomicin MICs were slightly higher among the highly related ribotype (RT) 027 (*n* = 22) and RT198 (*n* = 8) isolates (GM MIC of 0.08 mg/liter for both) than for all isolates (0.04 mg/liter), this was not statistically significant (Kruskal-Wallis *P* = 0.86 and 1.00, respectively). Conversely, the fidaxomicin MICs were statistically significantly lower among RT001 isolates (Kruskal-Wallis *P* = 0.0001), with a GM MIC of 0.01 mg/liter, reflecting previous results (5, 7, 8). The SMT19969 MICs for RT027 (GM = 0.11 mg/liter) and RT017 (GM = 0.12 mg/liter) isolates were slightly elevated above those for all isolates, but this was not statistically significant (Kruskal-Wallis *P* = 0.30 and 0.29, respectively). Ribotypes 027, 198, and 017 were associated with multiple antimicrobial resistance in a previous study (5). The slightly elevated fidaxomicin and SMT19969 GM MICs observed against selected ribotypes are unlikely to have clinical significance, given the high intraluminal gastrointestinal (GI) concentrations of both agents (9, 10). The GM metronidazole MICs were also slightly higher among RT027 and RT198 (1 mg/liter for both) isolates than those for all isolates (0.4 mg/liter), in line with previous observations (5, 8). However, despite low gut concentrations, metronidazole treatment failure has not been linked to decreased susceptibility to this agent (8).

There was a significant correlation between increased CRS and increased SMT19969 MICs (Pearson's product-moment correlation *r* = 0.33; *P* = 0.004), metronidazole MICs (*r* = 0.27; *P* = 0.004), and, to a lesser degree, fidaxomicin MICs (*r* = 0.25; *p* = 0.01), but no such correlation for vancomycin (*r* = 0.12; *P* = 0.21). A comparison of susceptibilities by ribotype in this study would inevitably contain bias, given that the selection criteria were based on the resistance phenotypes; however, it is worth noting that the C. difficile isolates with the highest MICs of SMT19969 belonged to ribotypes noted for resistance to multiple antimicrobials.

Isolates were selected to represent a broad range of antimicrobial resistance phenotypes. The results for metronidazole, vancomycin, fidaxomicin, rifampin, moxifloxacin, clindamycin, chloramphenicol, and tigecycline largely reflected those previously determined (5), with evidence of high-level resistance to rifampin, moxifloxacin, clindamycin, and chloramphenicol (Table 1). Imipenem resistance was low (3.7%), and reduced susceptibility to tigecycline was very rare (<1%). Most isolates were linezolid susceptible (85.3%), but, unexpectedly, 13.8% showed high-level resistance (>16 mg/liter). These isolates belonged to RT001 (7 of 22), RT017 (2 of 7), RT027 (4 of 22), and RT356 (2 of 4). There was location clustering in RT001, with 3 linezolid-resistant isolates from the same geographical location. These isolates also showed resistance to clindamycin and in some cases chloramphenicol (Table 2). A recent publication also described linezolid resistance among RT001, RT078, RT126, and RT017 isolates from Spain (11). The authors demonstrated the presence of the multidrug resistance gene, *cfr*, in isolates showing high-level resistance to chloramphenicol, erythromycin, clindamycin, and linezolid from RT017, RT078, and RT126. This was linked to a mobile genetic element, Tn*6218*, indicating the possibility of transmission between strains. They were unable to demonstrate the presence of *cfr* in the remaining RT001 isolates that showed lower chloramphenicol MICs, suggesting that other resistance mechanisms are involved (11). There is likely to be more than one etiology for the linezolid resistance seen in the isolates tested here, given the phenotypes displayed. It is interesting to note that all of the linezolid-resistant isolates also displayed moxifloxacin resistance. This combination was associated with higher cadazolid MICs (2- to 4-fold higher than those of susceptible isolates with resistance to either moxifloxacin or linezolid) (12) and may not be unexpected since cadazolid is an oxazolidinone-fluoroquinolone hybrid molecule. However, its clinical significance is unknown, given the high fecal cadazolid concentrations achieved (13).

**TABLE 2:** High-level resistance to linezolid among PCR ribotypes 001, 017, 027, and 356

Ribotype	MIC (mg/liter)											
	SMT19969	Fidaxomicin	Metronidazole	Vancomycin	Rifampin	Moxifloxacin	Clindamycin	Imipenem	Chloramphenicol	Tigecycline	Linezolid	Ceftriaxone
001	0.06	015	1	0.5	0.002	32^*a*^	128^*a*^	2	256^*a*^	0.03	16	64^*a*^
001	0.03	0.008	1	0.5	0.001	16^*a*^	128^*a*^	8^*b*^	16^*b*^	0.03	32^*a*^	128^*a*^
001	0.06	0.015	1	2	0.002	16^*a*^	64^*a*^	4	32^*a*^	0.03	32^*a*^	128^*a*^
001	0.125	0.008	0.5	0.5	0.002	16^*a*^	128^*a*^	4	16^*b*^	0.03	32^*a*^	128^*a*^
001	0.125	0.008	1	0.5	0.002	16^*a*^	128^*a*^	4	16^*b*^	0.03	32^*a*^	128^*a*^
001	0.125	0.008	1	0.5	0.002	16^*a*^	128^*a*^	4	16^*b*^	0.03	32^*a*^	128^*a*^
001	0.06	0.008	1	0.5	0.002	16^*a*^	128^*a*^	2	16^*b*^	0.03	32^*a*^	64^*a*^
017	0.125	0.06	0.25	0.5	0.001	32^*a*^	128^*a*^	4	16^*b*^	0.06	32^*a*^	32^*b*^
017	0.25	0.06	0.125	0.5	32^*a*^	32^*a*^	128^*a*^	4	64^*a*^	0.06	32^*a*^	64^*a*^
027	0.06	0.06	1	1	32^*a*^	16^*a*^	8^*a*^	8^*b*^	4	0.03	16^*a*^	128^*a*^
027	0.25	0.06	1	1	32^*a*^	32^*a*^	8^*a*^	8^*b*^	4	0.03	16^*a*^	64^*a*^
027	0.25	0.06	1	0.5	32^*a*^	32^*a*^	128^*a*^	4	64^*a*^	0.03	32^*a*^	128^*a*^
027	0.25	0.125	1	0.5	0.002	32^*a*^	128^*a*^	4	64^*a*^	0.03	32^*a*^	64^*a*^
356	0.03	0.06	1	1	32^*a*^	32^*a*^	8^*a*^	8^*b*^	4	0.03	16^*a*^	256^*a*^
356	0.06	0.06	0.5	2	32^*a*^	32^*a*^	16^*a*^	8^*b*^	8	0.03	16^*a*^	256^*a*^

a Isolate that is resistant.

b Isolate that is intermediate.

Only 6% of isolates were susceptible to ceftriaxone (GM MIC of 58.2 mg/liter) according to the breakpoints used (14). The highest levels of resistance (>128 mg/liter) were seen in RT356 isolates (all) and in 5 of 10 RT018 isolates, which are closely related. Ribotype 356 is exclusive to Italy, and all 5 RT018 isolates showing MICs of >128 mg/liter were also from this location. This high-level ceftriaxone resistance adds to the previously reported multidrug resistance in RT018 and RT356 isolates from Italy (5). Two of the RT356 isolates also showed intermediate imipenem resistance (Table 2).

In summary, SMT19969 was highly active against more than 100 isolates displaying different antimicrobial resistance phenotypes. There was no evidence of SMT19969 or fidaxomicin resistance, but some evidence of modestly higher SMT19969 and fidaxomicin MICs among ribotypes previously noted for multiple antimicrobial resistance. Linezolid resistance was more prevalent than expected and was also associated with ribotypes noted for multidrug resistance phenotypes. High-level ceftriaxone resistance was found in multiresistant RT018 and RT356 isolates from Italy.
